# Living Invisible: HTLV-1-Infected Persons and the Lack of Care in Public Health

**DOI:** 10.1371/journal.pntd.0001705

**Published:** 2012-06-12

**Authors:** Karina Franco Zihlmann, Augusta Thereza de Alvarenga, Jorge Casseb

**Affiliations:** 1 Federal University of São Paulo, São Paulo, Brazil; 2 Public Health Faculty, University of São Paulo, São Paulo, Brazil; 3 Laboratory of Medical Investigation LIM-56/Faculty of Medicine, Institute of Tropical Medicine of São Paulo, University of São Paulo, São Paulo, Brazil; George Mason University, United States of America

## Abstract

**Introduction:**

Human T-cell lymphotropic virus type 1 (HTLV-1) infection is intractable and endemic in many countries. Although a few individuals have severe symptoms, most patients remain asymptomatic throughout their lives and their infections may be unknown to many health professionals. HTLV-1 can be considered a neglected public health problem and there are not many studies specifically on patients' needs and emotional experiences.

**Objective:**

To better understand how women and men living with HTLV-1 experience the disease and what issues exist in their healthcare processes.

**Methods:**

A qualitative study using participant observation and life story interview methods was conducted with 13 symptomatic and asymptomatic patients, at the outpatient clinic of the Emilio Ribas Infectious Diseases Institute, in Sao Paulo, Brazil.

**Results and Discussion:**

The interviewees stated that HTLV-1 is a largely unknown infection to society and health professionals. Counseling is rare, but when it occurs, focuses on the low probability of developing HTLV-1 related diseases without adequately addressing the risk of infection transmission or reproductive decisions. The diagnosis of HTLV-1 can remain a stigmatized secret as patients deny their situations. As a consequence, the disease remains invisible and there are potentially negative implications for patient self-care and the identification of infected relatives. This perception seems to be shared by some health professionals who do not appear to understand the importance of preventing new infections.

**Conclusions:**

Patients and medical staff referred that the main focus was the illness risk, but not the identification of infected relatives to prevent new infections. This biomedical model of care makes prevention difficult, contributes to the lack of care in public health for HTLV-1, and further perpetuates the infection among populations. Thus, HTLV-1 patients experience an “invisibility” of their complex demands and feel that their rights as citizens are ignored.

## Author Summary

Human T-cell lymphotropic virus type 1 (HTLV-1) infection is commonly confounded with Human Immunodeficiency Virus (HIV) infection and it is unknown to many health professionals. It is endemic in many countries and there is no effective treatment available. Although a few individuals have severe symptoms, most patients remain asymptomatic throughout their lives. Further, HTLV-1 is considered a neglected public health problem and limited studies cover specific patients' needs and emotional experiences. To better understand how women and men living with HTLV-1 experience the disease and what issues exist in their healthcare processes, we conducted a qualitative study of both symptomatic and asymptomatic patients at an outpatient clinic at the Emílio Ribas Infectious Diseases Institute in São Paulo, Brazil. We found that the main focus of health staff was on illness risk, but not identifying infected relatives and preventing new infections. This point of view, ultimately neglected patients' complex demands, and overshadows the prevention of new infections and contributes to the lack of care in public health for HTLV-1 infected subjects. Furthermore, this perpetuates the infection among these populations and the patients experience an “invisibility” of their specific needs, such as reproductive rights and feel that their rights as citizens are ignored.

## Introduction

The human T-cell lymphotropic type 1 (HTLV-1) [1] infection causes a life-long infection for infected subjects. HTLV-1 is endemic in various parts of the world, including Japan and countries in Africa, the Caribbean and South America [2]. It has been estimated that over 10 million individuals are infected with HTLV-1 [3], but most infected persons are asymptomatic and probably are not aware of their serological status. In addition, asymptomatic individuals may still infect sexual partners or offspring.

The most common diseases associated with HTLV-1 infection are adult T-cell leukemia/lymphoma (ATL) and HTLV-1-associated myelopathy/tropical spastic paraparesis (HAM/TSP) [4]. No accurate case statistics exist for ATL or HAM/TSP because these diseases are not reportable by the World Health Organization (WHO). Despite several publication reviews on pathogenesis or molecular biology of HTLV-1 [5], [6], few studies have addressed treatments for HTLV-1 or the psychological issues related to having the disease. Therefore, HTLV-1 infection is considered, and was recently reported, as a neglected disease [7].

Brazil has received worldwide recognition for the country's public health policies, especially with respect to how the nation has addressed the pandemic of HIV/AIDS [8]. Such success against HIV/AIDS was possible only because Brazil's constitution recognizes and guarantees healthcare as a right of every citizen and the country's public health ministry provides a multidisciplinary prevention program and free medication for HIV/AIDS. [8]. However, such public health policies have not been applied to HTLV-1 infection, which is not even listed as an infectious disease that should be addressed by public health action. Thus, HIV/AIDS may overshadow the problem of HTLV-1 in Brazil, and consequently, HTLV-1 has become essentially unknown to most of health professionals and should be considered a public health problem in Brazil [9].

Few HTLV-1 epidemiological studies have been conducted in the general population, but the average prevalence of HTLV-1/2 among donor blood banks is 0.46% nationwide [10] and one few based on study population showed prevalence of 1.7% in Salvador, with 40000 individuals are estimated to be infected by HTLV-1 [11]. The highest prevalence of HTLV-1 has been recorded in São Luís city in Maranhão, with 10.0/1000 and Salvador in Bahia with 9.4/1000 [11]. However, other cities may present lower prevalence despite geographical proximity, which indicates heterogeneous distribution and cluster pattern [12].

In 1993, it was recommended by ministry of health the mandatory tests for hepatitis C and HTLV-1/2 in blood banks. In 2002, Brazil also instituted a policy which provided free testing of HIV/AIDS and syphilis during prenatal care visits as an approach to preventing mother-to-child-transmission (PMTCT). However, the same policy was not provided for HTLV-1 infection and the Brazilian Ministry of Health only published the Guide Recommendations on HTLV Management in 2003, without update [13].

From a public health perspective, prevention of HTLV-1 is crucial, especially because HTLV-1 is a life-long infection and is currently incurable and untreatable. The health field recognizes that disease prevention requires strategies beyond strictly medical approaches. Thus, the aim of this study was to identify perceptions of HTLV-1 by women and men living with this infection, and to better understand their families, subjective issues, needs and how they cope with health care.

## Materials and Methods

The present study was conducted by using participant observation methods [14] in the HTLV outpatient clinic at the Emílio Ribas Infectious Diseases Institute in São Paulo, Brazil from June 2006 to April 2008. This is centenary national public referral hospital specializing in the diagnosis and treatment of individuals with infectious diseases.

Using a thematic script and in parallel an in-depth interviews with 13 individuals (11 women and two men) diagnosed with HTLV-1 infection and without co-infections (see Table 1). Subjects were selected and interviewed during the regular visit to the HTLV clinic. This convenience sample tried to cover the most part of situations such as gender, presence of symptoms, serological couple status and so on [14].The questions of the thematic script were about the subjects history of life, their knowledge and experiences related to the HTLV-1 infection, related diseases and family perception of HTLV-1 serological status disclosure.

**Table 1:** Socio-demographic characteristics and HTLV-1 infection data for interviewed patients.

Name	Age (years)	Marital status	Education (years)	Symptomatic	Children	Relatives tested	Modes of transmission
Lidia	34	single	8	yes	none	no	MTCT
Ana	27	married	11	yes	1	no	MTCT
Angélica	26	divorced	5	yes	3	no	blood transfusion
Alba	27	married	6	yes	none	no	MTCT
Marta	31	married	16	no	none	no	unknown
Carolina	61	widower	11	no	2	no	unknown
Silvia	42	married	11	no	3	no	unknown
Alice	23	married	11	no	none	no	MTCT
Fabiana	27	married	16	no	none	no	unknown
Maria	27	married	11	no	pregnant	yes	sex
Maria Rita	27	married	11	no	1 pregnant	no	unknown
Victor	47	divorced	5	yes	3	no	unknown
Walter	33	married	11	yes	wife pregnant	no	MTCT

Data were analyzed by using life narratives from each subject and constructed content categories from these narratives [15]. The reported names of participants are fictitious in order preserve their privacy and identities.

The study was approved by the Ethical Research Board Committee - CAPPesq (n. 297/06) and the Emílio Ribas Institute of Infectious Diseases Ethical Committee (n. 34/06). The volunteers signed an informed consent form that faithfully follows the resolution of the national Ministry of Health (CNS 196/96) and the Declaration of Helsinki.

## Results and Discussion

### The invisibility as a central category of analysis

While HTLV-1 infection is frequently “invisible” in a literal way, this article aimed to highlight the disease's symbolic invisibility from patients' perspectives and life experiences, as well as patients' relations with healthcare providers.

### The view of people with HTLV-1: feeling invisible from being diagnosed with an “invisible” disease

The HTLV-1 diagnosis is frequently a doubts and fear moment in primary medical care settings. While recalling their original diagnosis, subjects reported an initial shock followed by some feelings of denial (e.g., “it is not true”) or hopefulness for a “cure.” In addition, many participants thought that their illness was HIV infection, which has been similarly reported by Guiltinan (1998) [16]. This erroneous observation was also found in some health professionals speeches. Subsequently, this confusion illustrated the lack of knowledge about HTLV-1 among health professionals and society in general. Furthermore, subjects reported insecurity and doubts about the abilities of their primary healthcare providers; however, patients indicated that these problems were better addressed when they were referred to specialized care.

Interestingly, many subjects reported that “it was better not to know about HTLV” and “not to think about HTLV”. These observations indicated the use of denial as a defense mechanism. Subjects also reported fear of disclosure and that their HTLV-1 diagnoses would be disrupted family relations. Consequently, this perception leads subjects to hide their HTLV-1 diagnosis from everyone, as a “secret.” Such difficulties have also been observed in people living with HIV/AIDS [17], especially as related to their fears of disease stigma [18]. Subjects noted “justification[s]” for keeping their disease status secret, reasoning that “family members are healthy, symptomless and therefore there is no need worry” and they did not want to cause emotional suffering for relatives. These statements illustrated elements of a singular logic underlying the desire for HTLV-1 concealment among family members.

Regarding this “secret,” the thoughts and perceptions between subjects and health providers seem connected. Subjects and providers understood the absence of HTLV-1 symptoms as “normal”, in consequence HTLV-1 infection remained “invisible” because they are focused on risk of sickness. This observation has important implications for understanding the lack of diagnosis among partners and family members.

*“Living with HTLV is normal for me. I feel nothing, do not take any medication. For me, HTLV does not bring any problem, feel anything. How can I bring my mother to be tested, disturbing everyone?” (Silvia, 42 years old, married, asymptomatic, has three children).*

*“I prefer not to think and not bring my children to be tested. I think it is better this way. I'm dealing with HTLV. I do not want to mention it. If I do this, it can mess with my children minds”. (Victor, 33 years old, married, symptomatic - HAM/TSP)*

Except for Maria, all subjects indicated that someone in their family circle (i.e., sexual partners, relatives or children) should be tested for HTLV-1 infection but had not been tested yet. This situation illustrated how poor HTLV-1 disclosure among family members has negative consequences. In Maria's case the lack of disclosure was the possible cause of her infection, because Maria's husband, Walter, was infected by his mother, and hidden his HTLV-1 infection from her. Her husband, Walter, did not reveal his diagnosis to his wife because he thought they were both healthy. Although his wife was pregnant (30 weeks), Walter only sought medical attention when he began to have symptoms of HAM/TSP. Only on this time, his health team requested to his pregnant woman to be tested for HTLV-1 infection.

The symptomatic condition further expanded upon these issues, as patients experience changes in their daily activities, self-image and social lives. The progressive disability associated with symptomatic HTLV-1 infection limited these patients' lives, as illustrated by the sentences below.

*“After I developed this disease my husband told me that I'm not good for anything anymore. As much as we do not want to remember this, this prejudice keeps reminding us of the disease” (Angelica, 26 years old, divorced, HAM/TSP, has three children).*

*“People already have a bias to get close because I was not walking well. If you have a problem, they say that you are no different. They say that you are the same. But it is not true! You have a huge problem because you have a difficulty to walk. Imagine if you say that you have an infectious disease” (Lidia, 34 year old, single, HAM/TSP, has no children).*

The suffering and mobility limitations constitute “narcissistic injuries” [19], which is related to the self-steam in deeper emotional level or in unconscious influence. This situation generated intense anxiety, defense mechanisms and required coping strategies among these patients. In this context, it is understandable that persons living with HTLV-1 had difficulties, which may explain reluctance to share their experiences with their family.

Subjects report that their perceptions and experiences changed when their family members are symptomatic. HTLV-1 becomes present in subjects' lives through the symptoms of their family, and the need to care for these members may trigger additional emotional coping mechanisms.

In cases where subjects know that they were infected by their parents, HLTV-1 infection is viewed as a type of “family heritage” that the subject must “carry for life.” This knowledge may be very painful emotionally, as demonstrated by Alba and Walter:

*“I can not accept it. I thought, why my story is so painful? HTLV is one heritage! I kept myself a virgin for my future husband. I did not get anything financially from my family but I inherited a disease” (Alba, 27 yeas old, married, HAM/TSP, has no children).*

*“HTLV is a painful thing because it's something we will carry for life”. (Walter, 33 years old, married, HAM/TSP).*

This lack of disclosure to partners and family on HLTV-1 diagnosis could be explained by poor information regarding infection transmission; however, the speeches revealed an appropriate understanding of transmission modes. On the other hand, some subjects had doubts how they became infected. These subjects were not willing to search for this information, which illustrates their difficulties in dealing with the HLTV-1 diagnoses.

Invisibility among persons living with HTLV-1 may have meaning, especially because patients have reported feeling stigmatized and socially disadvantaged (e.g., “discarded” or “limited”) since receiving their diagnoses [17]. As patients became aware of their HTLV-1 infection, they were forced to reorganize their priorities. Reproductive decisions and sexuality were especially pronounced, as Alba's speech illustrated.

*“Having a child would be a burden of conscience. I would try to do something to no longer generate another discarded person in my family. Another contaminated one in the family.” (Alba, 27 years old, married, HAM/TSP, has no children, her father also has HAM/TSP).*

### Health professionals and their healthcare practices from the subjects' perspective: hegemonic conceptions and practices in HTLV-1 prevention

According to subjects, primary health teams are generally unaware of HTLV-1 and did not investigate or recognize the health problems associated with the infection and disease. Subjects reported a poor access to pre- and post-test counseling for HTLV-1 infection. In fact, an author showed that a person can be tested and remained without access to specific information and follow up [16]. Even when subjects had some guidance in counseling, health professionals focused on low risk of developing symptoms and failed to emphasize the risk of transmission to partners or to provide orientation regarding reproductive decisions and maternal-infant transmission risks.

Most interviewers reported they were the only sources of information about HTLV for the health teams, which created uncertainty concerning the teams' medical knowledge. This finding leads to a paradoxical question on healthcare for HTLV: how can a doctor inform and treat patients when himself does not know HTLV? Maria and Maria Rita's speeches, who were both pregnant, illustrated this paradox.

*“The doctor asked me what HTLV infection is. But I did not know it either. Even doctors do not know what it is, can I imagine? They said ‘I cannot say anything. I will first study and then I will see what I have to do’” (Maria, 27 years old, married, asymptomatic, pregnant, one child who is eight years old).*

*The obstetrician who provided prenatal care explained the following to Maria Rita: “You have a virus like AIDS. I do not know if this disease can be transmitted to the baby. I do not know anything about this virus”. Another medical staff member, during an ultrasound exam, told her: “If the virus has infected the baby, apparently it did not affect anything. But I do not know anything about this virus”.*

The “invisibility” of HTLV-1 infection should not be a problem for symptomatic patients such as HAM/TSP and ATL cases. However, this study revealed that symptomatic patients had to make “pilgrimages” to several specialists as they searched for a diagnosis. In fact, HAM/TSP patients experienced an average of eight years seeking a diagnosis [20], extending up to 11 years in some cases [21]. Being symptomatic, or considered a “patient,” should generate the search to identify the cause of symptoms. For HAM/TSP diagnosis, the HCP must have the knowledge and make the differential diagnosis, which firstly requires HTLV-1 serology. Unfortunately, HTLV-1 serology is not always available, even in institutions which are specialized in treating mobility problems [21].

Interviewers and medical staff were concerned on the risk of disease development, which is considered “low” for both parties. Healthcare providers and HTLV-infected subjects frequently do not realize that asymptomatic individuals may transmit the virus to partners or offspring. Consequently, preventive processes may become ineffective.

In conclusion, there appeared to be an underlying logic for the “invisibility” in people living with HTLV-1. Even when individuals had access to necessary information regarding the disease, they frequently denied the situation. In consequence, there were implications for self-care and understanding the need for testing family members for HTLV-1 infection.

As people living with HTLV-1 struggle to cope with this health problem, it is possible to observe that these difficulties were logically articulated through the hegemonic healthcare approach. This hegemonic conception of health care is referred to major paradigm in health focused at the biomedical approach. Nowadays, it has been questioned by the researchers at the public health field to contrast this model to the bio-psych-social model which aims the health care in the entire complexity. The biomedical perspective does not encourage prevention activities and health promotion [22]. The current hegemonic context, a true “poor-care” exists toward people living with HTLV-1, further contributing to the perpetuation of this neglected disease by society in endemic areas.

Beyond the “invisibility” of HTLV-1, there is an “invisibility” of the subjects living with the infection or disease, their individual difficulties and idiosyncrasies [23], and their rights as citizens. In order to bring HTLV-1 from “invisibility” to “visibility,” the paradigm of standard healthcare should focus on effective preventive practices, which may consider patients' complex demands. Reports from subjects also indicated the need for studies on sexuality and reproductive decisions among people living with HTLV-1 infection, as these issues had been shown to directly influence prevention efforts.
